# Novel treatment of severe combined immunodeficiency utilizing *ex-vivo* T-cell depleted haploidentical hematopoietic stem cell transplantation and CD45RA+ depleted donor lymphocyte infusions

**DOI:** 10.1186/s13023-016-0385-3

**Published:** 2016-01-15

**Authors:** Nicholas Brodszki, Dominik Turkiewicz, Jacek Toporski, Lennart Truedsson, Josefina Dykes

**Affiliations:** Children’s Hospital, Skåne University Hospital, Lund, Sweden; Department of Laboratory Medicine, Section of Microbiology, Immunology and Glycobiology, Lund University, Lund, Sweden; Department of Laboratory Medicine, Section of Haematology and Transfusion Medicine, Lund University, Lund, Sweden

**Keywords:** Pediatric, Immunodeficiency, Hematopoietic stem cell transplantation, Lymphocytes, Donor lymphocyte infusion, Haploidentical, T cell depletion

## Abstract

**Background:**

Allogeneic hematopoietic stem cell transplantation (HSCT) is the only curative treatment available for severe combined immunodeficiency (SCID); although, there is a high incidence of severe infections and an increased risk of graft-versus host-disease (GvHD) with HSCT. Early intervention is a crucial prognostic factor and a HLA-haploidentical parental donor is often available. Haploidentical HSCT protocols utilizing extensively *ex vivo* T-cell depleted grafts (CliniMACs system) have proven efficient in preventing GvHD, but cause a delay in early T-cell recovery that increases the risk of viral infections. Here, we present a novel approach for treating SCID that combines selective depletion of GvHD-inducing alpha/beta (α/β) T-cells from the haploidentical HSCT graft with a subsequent donor lymphocyte infusion (DLI) enriched for CD45RO+ memory T-cells.

**Results:**

Our patient was diagnosed with SCID (T-B + NK+ phenotype). At 9 months of age, he received a T cell receptor(TCR)α/β-cell depleted graft from his haploidentical mother, following a reduced intensity conditioning regimen with no additional GvHD prophylaxis. Engraftment was rapid with complete donor chimerism and no signs of GvHD. However, at 12 weeks post HSCT, the patient was still T-cell lymphopenic with clinical symptoms of multiple severe viral infections. Consequently, therapeutic DLIs were initiated for enhanced anti-viral immunity. The patient was treated with CD45RA+ depleted haploidentical maternal donor lymphocytes enriched from unmobilized whole blood, and a total T-cell dose of no more than 25 x10^3^ CD3+ cells/kg with >99.9 % purity of CD3 + CD45RO+ memory T-cells was transferred. Following the DLI, a prompt increase in CD3 + CD4+ and CD3 + CD8+ counts was observed with a subsequent clearance of viral infections. No acute or chronic GvHD was observed.

**Conclusions:**

Automated depletion of CD45RA+ naïve T-cells from unmobilized whole blood is a simple and rapid strategy to provide unmanipulated DLIs, with a potentially broad repertoire of pathogen specific memory T-cells. In the haploidentical setting, CD45RA+ depleted DLIs can be safely administered at low T-cell doses for efficient enhancement of viral immunity and limited risk of GvHD. We demonstrate the successful use of this approach following TCR-α/β-cell depleted HSCT for the treatment of SCID.

## Background

Severe combined immunodeficiency (SCID) is a rare disorder defined by a profound developmental or functional defect of T lymphocytes. If unrecognized, it can cause death during the first years of life, due to the life-threatening increased susceptibility to infections [1] therefore early detection and treatment of SCID, before infections become overwhelming, optimizes survival [2–4]. Today, most forms of SCID are treated with hematopoietic stem cell transplantation (HSCT) and if HSCT is not successful and genetic treatment is not an option, the alternatives are few [5].

For patients lacking a human leukocyte antigen (HLA)-matched sibling or unrelated stem cell donor or when there is an urgent need for a HSCT, an HLA-haploidentical parental donor is often readily available [6]. Given the major HLA donor-recipient disparities in the haploidentical setting, measures are required to prevent the occurrence of alloreactive responses, i.e. graft rejection or severe graft-versus host-disease (GvHD). The extensive *ex-vivo* depletion of donor T-cells (CliniMACS system) has proven efficient in preventing GvHD [6, 7], but is inevitably coupled to a delay in early T-cell recovery and, thus, to an increased risk of viral infections [6–9]. Recent efforts to balance the risk of GvHD against that of delayed immune reconstitution include the selective depletion of GvHD-inducing alpha/beta (α/β) T-cells while retaining potentially beneficial gamma/delta (γ/δ) T-cells in the haploidentical graft [10, 11].

Unselected donor lymphocyte infusions (DLIs), which are frequently used as a “tool” to boost anti-viral immunity post-transplant, harbor a significant risk of inducing severe GvHD in the haploidentical setting [6, 7]. GvHD-inducing cells reside mainly in the CD45RA+ naïve T-cell population whereas viral-specific memory T-cells are predominantly CD45RA-negative [12]. Thus, the depletion of CD45RA+ T cells from DLIs may provide a potentially broad repertoire of donor-derived viral-immunity with a limited risk of GvHD [13, 14].

Herein, we report on the successful treatment of SCID by combining T cell receptor (TCR)-α/β-cell depleted haploidentical HSCT with CD45RA+ depleted DLI for an antiviral boost.

## Methods

### Patient

The male patient was born at full term, after a normal pregnancy, as the third child of Afghanistani related parents. Both parents and the siblings are healthy. He had his first upper respiratory tract infection (URTI) at the age of 2 weeks and at the age of 1 month, he was admitted for the first time to a pediatric ward for 2 weeks with coughing, low grade fever and hoarseness. In the following 3 months, he was followed regularly due to coughing, hoarseness and failure to thrive. At the age of 4 months, he was infected with chicken-pox. Three weeks after the onset of the infection, new blisters were still forming and varicella keratitis developed. The unusually severe course of the infection and the failure to thrive warranted investigation for primary immunodeficiency disorder (PID), in conformity with our protocol [15].

### Investigations

Routine blood investigations during his first 4 months of life showed normal leukocyte and neutrophil counts on several occasions. Total lymphocytes were measured during the first admission at 1 month of age, but the low value of 0.6 (ref: 3–8.4 × 10^9^/L) was attributed to an upper respiratory tract infection (URTI) and was followed up only at the beginning of the chicken-pox infection when the level was 6.2x10^9^/L. Other laboratory tests, including electrolytes, ALT, AST, GT, pancreatic amylase, bilirubin, organic acids, amino acids, and the thyroid hormone status, were normal. At the time point of PID investigation, he was anemic (hemoglobin: 81 g/L) with normal platelets and a total lymphocyte count of 2.2x10^9^/L. The immunological investigation revealed very low T-cell counts, confirming a T-B + NK+ SCID phenotype. The cultures taken at this point showed multiple infections: positive *Staphylococcus aureus* blood culture, cytomegalovirus (CMV), and varicella virus DNA positivity in blood, varicella DNA in the cerebrospinal fluid, *Pneumocystis jiroveci* and coronavirus NL63 on throat swabs, rotavirus in feces, and high serum beta-d-glucan.

### Genetic analysis

Genomic DNA was prepared from blood collected in EDTA, fragmented to an average length of 300 bp, and exome enriched using the SureSelect XT Human All Exon v5 technology (Agilent). Sequencing was carried out to an average coverage of 150-fold using PE 2x100 bp sequencing (Illumina HiSeq 2500). The bioinformatic analysis was carried out using the Mutation Identification Pipeline (MIP) [16]. Results were presented in an interactive browser-based visualization tool (Scout), developed in-house (Science for Life Laboratory, Solna, Sweden). The clinical interpretation of the results was restricted to a predefined set of 233 genes known to be involved in primary immunodeficiencies.

Sequence changes in exons and exon-intron boundaries were analyzed. Only rare variants (allele frequency <0.01) were considered. For recessive disease genes, at least two variants were required. The analysis was not designed to detect structural rearrangements or copy number alterations. The exon sequencing, restricted to the predefined set of 233 immunodeficiency genes, did not identify any low-frequency variants that could account for the patient’s clinical presentation. A homozygous missense variant (Val131Phe) in the *CD3G* gene, was identified, but it received a low rank score due to its reported high frequency of 0.21 in the 1000 Genomes database and was, therefore, considered a natural variant. Thus, no known genetic defect was identified.

### Donor selection

Considering the low numbers of lymphocytes and the life-threatening infections, the decision was made to perform a HSCT. The parents provided written informed consent for this procedure. No HLA matched siblings were available and the probability of finding a matched unrelated donor was considered low given the patient´s ethnical origin. Consequently, the choice was made to select a HLA-haploidentical parental donor. The patient and his parents were investigated according to our institutional protocol for haploidentical HSCT, including high resolution allelic typing for HLA- A*, -B*, -C,* DRB1*, DQB1*, BPB1* (PCR-sequence specific primers [SSP], Olerup SSP AB), HLA-donor specific antibodies (DSA) (LabScreen Singel Antigen, Luminex platform, One Lambda, Thermo Fisher), ABO-blood grouping and CMV-serology. The mother was selected as the donor based on the outcome of the pre-transplant investigation, and this selection was further supported by the reported higher survival rate in haploidentical HSCT patients grafted from the mother compared with recipients of paternal grafts [17]. The HLA-typing confirmed patient and donor haploidentity with a DRB1*, DQB1*, and DPB1* allelic match in the host-versus-graft direction. No DSA were detected in the patient´s serum. The patient and the donor were matched for ABO-blood group and were both CMV positive.

### Transplant protocol

The patient was conditioned with a myeloablative regimen, according to guidelines [18] regarding HSCT for primary immunodeficiencies with a haploidentical family donor in patients older than 3 months. However, due to his very poor health status, we decided to use a reduced intensity regimen conditioning. This consisted of i.v. anti-thymoglobuline (ATG Fresenius ®) given from day -9 to -6 (first day 1 mg/kg bodyweight and then 10 mg/kg bodyweight), fludarabine (40 mg/m^2^/day, from day -7 to -4), thiotepa (5 mg/kg twice a day; day -3), and melfalan (70 mg/m^2^ day -2 to -1). The sole form of GvHD prevention was the immunomagnetic depletion of TCR-α/β + cells from the graft. Prophylaxis against post-transplant Epstein–Barr virus-associated lymphoproliferative disease (EBV-PTLD) consisted of one dose of rituximab (375 mg/m^2^) given at day +1. No post-transplantation pharmacological immunosuppression was used.

### Graft engineering

Peripheral blood progenitor cells (PBPCs) of the haploidentical maternal donor were mobilized by granulocyte-colony-stimulating factor (G-CSF, 10 μg/kg/day) and collected by leukapheresis (Cobe Spectra, Terumo BCT) on day 4 of G-CSF administration. Collected PBPCs were processed under clean room conditions with the CliniMACS system (Miltenyi Biotech), according to the manufacturer´s protocol. The cells were sequentially labelled with TCR-α/β-Biotin reagent and CliniMACS anti-Biotin reagent, and processed with the CliniMACS device using the D3.1 program with the Depletion Tubing Set (DTS). The graft cell contents were determined by flow cytometric analysis (FACS, Canto II, Becton Dickinson [BD]), using antibodies against CD45, CD34, CD14, CD19, CD56, CD3, TCR-α/β and TCR-γ/δ with 7-AAD as a viability marker (BD). According to our institutional protocol for haploidentical HSCTs, the target cell dose of the TCR-α/β-cell depleted graft was set to >10 × 10^6^ CD34+ cells/kg (minimum 5 × 10^6^ CD34+/kg) and <25 × 10^3^ TCR-α/β + cells/kg (maximum 1 × 10^5^ TCR -α/β+ /kg).

### Manufacturing of donor lymphocytes

A bag of unmobilized whole blood was drawn from the donor. Utilizing a closed blood bag system, whole blood was separated by density centrifugation to obtain a leukocyte enriched cell fraction for further processing with the CliniMACS system. According to the manufacturer´s protocol, cells were labelled with CD45RA reagent and processed under clean room conditions with the CliniMACS device using the D3.1 program with the DTS. The cell contents of the CD45RA-depleted target fraction was assessed by FACS (Canto II, BD), using antibodies against CD3, CD4, CD8, CD45RO, and CD45RA with 7-AAD as a viability marker (BD).

### Post-treatment evaluation

Engraftment following HSCT was monitored by daily counts of leukocytes, neutrophils, and platelets. Immune recovery was assessed post HSCT and after DLI by FACS (Canto II, BD) of peripheral blood lymphocytes, using antibodies against CD3, CD4, CD8, CD19, CD56, TCR-α/β, TCR-γ/δ, CD45RA and CD45RO (BD). Donor myeloid and T-cell chimerism was monitored by DNA genotyping of short tandem repeat polymorphisms (PCR-STR, 3500 Dx Genetic Analyzer, Applied Biosystems). The patient was evaluated for signs of acute and chronic GvHD.

### Compliance with ethical guidelines

All procedures followed were in accordance with the ethical standards of the Helsinki Declaration of 1975, as revised in 2000. The Regional Ethical Review Board in Lund, Sweden advised us that signed consent from the patient’s parents was sufficient for publication of this case and the consent was obtained.

## Results

### Collection, depletion, and recovery

A single leukapheresis procedure provided 31 × 10^6^ CD34+ cells/kg recipient weight for subsequent TCR-α/β-cell depletion with the CliniMACS system. The log_10_ TCR-α/β-cell depletion rate was -5.0, corresponding to 0.005 % TCR-α/β + cells in the target fraction. The recovery of CD34+ cells and TCR-γ/δ + cells was 99 %. Cell viability post-sorting was 98 %. The TCR-α/β-depleted graft contained per kg of recipient weight 30.8 × 10^6^ CD34+ cells, 0.11 × 10^5^ TCR-α/β + cells, 29.7 × 10^6^ TCR-γ/δ + cells, 237.7 × 10^6^ CD19+ B-cells and 162.2 × 10^6^ CD56+ NK-cells and was transplanted in total to the patient. The donor had antibodies against CMV, VZV EBV and herpes simplex.

### Engraftment and immunological reconstitution

**Fig. 1:**
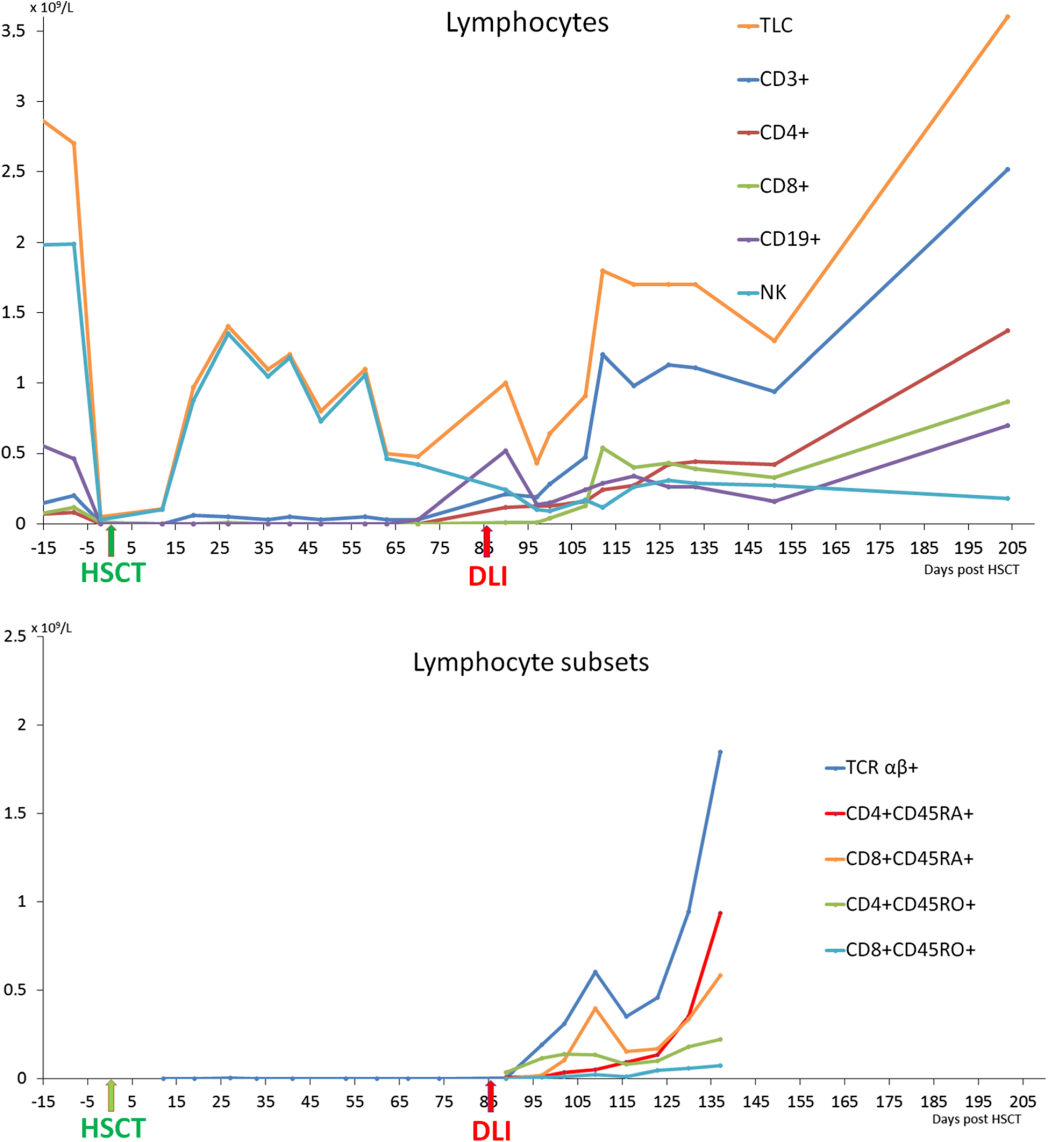
Total lymphocyte count (TLC) and concentrations of different lymphocyte subsets (indicated by CD positivity) over time in relation to hematopoietic stem cell transplantation (HSCT) and donor lymphocyte infusion (DLI). NK = Natural killer cells

Hematological recovery post HSCT was prompt with a white blood cell count >1 × 10^9^/L at day +10, neutrophils >0.5 × 10^9^/L at day +12, and thrombocytes >50 × 10^9^/L at day +13. Complete myeloid and T-cell donor chimerism was reached at day +19. As expected (16), NK-cells co-infused with the graft expanded rapidly, with normal peripheral counts from day + 14 (ref: 0.13–0.72 × 10^9^/L). However, despite the prompt three-linear hematological recovery, the patient remained profoundly T-cell lymphopenic (Fig. 1). ATG clearance vary between individuals and the efficacy of ATG varies between batches [19, 20]; therefore an explanation of the lymphopenia could be that since we used ATG in a relatively high dose, given timewise quite close to the grafting, this affected the graft, hence could be considered as additional in vivo T-cell depletion. Due to the persistent effect of rituximab, B-cell recovery was also markedly delayed.

### Donor lymphocyte infusions

A single unit of donor whole blood provided a surplus of CD3+ cells (62.2 × 10^6^/kg recipient weight) for subsequent CD45RA+ depletion using the CliniMACS system.

The log_10_ CD3 + CD45RA+ cell depletion rate was -3.8, corresponding to 0.0049 % residual CD3 + CD45RA+ cells in the target fraction. The recovery of CD3 + CD45RO+ cells was 51 %. Cell viability post-sorting was 96 %. The target fraction contained 12.6 × 10^6^ CD4 + CD45RO+ cells and 1.3 × 10^6^ CD8 + CD45RO+ cells per kg of recipient weight, with an increased CD4+/CD8+ ratio compared with the starting product (9.6 versus 2.1). Aliquots of CD45RA+ depleted products containing 25 × 10^3^ CD3+ cells/kg of recipient weight were prepared for fresh DLI and cryopreservation. The patient received a fresh CD45RA+ depleted DLI of 25 × 10^3^ CD3+ cells/kg from the original HSCT donor at day +84 post HSCT. At the time of DLI (day +84 post HSCT), peripheral blood CD3 + CD4+ and CD3 + CD8+ levels were <10 × 10^6^ /L, but reached 178 × 10^6^ /L and 111 × 10^6^/L, respectively, at 22 days following DLI. At 57 days after DLI, all T-lymphocyte subset counts were normalized (Fig. 1), corresponding to a continuously complete donor T-cell chimerism. The circulating T-cells were predominantly CD4 + CD45RA+ (935 × 10^6^ /L) and CD8 + CD45RA+ (586 × 10^6^ /L).

### Post-transplant morbidity

**Fig. 2:**
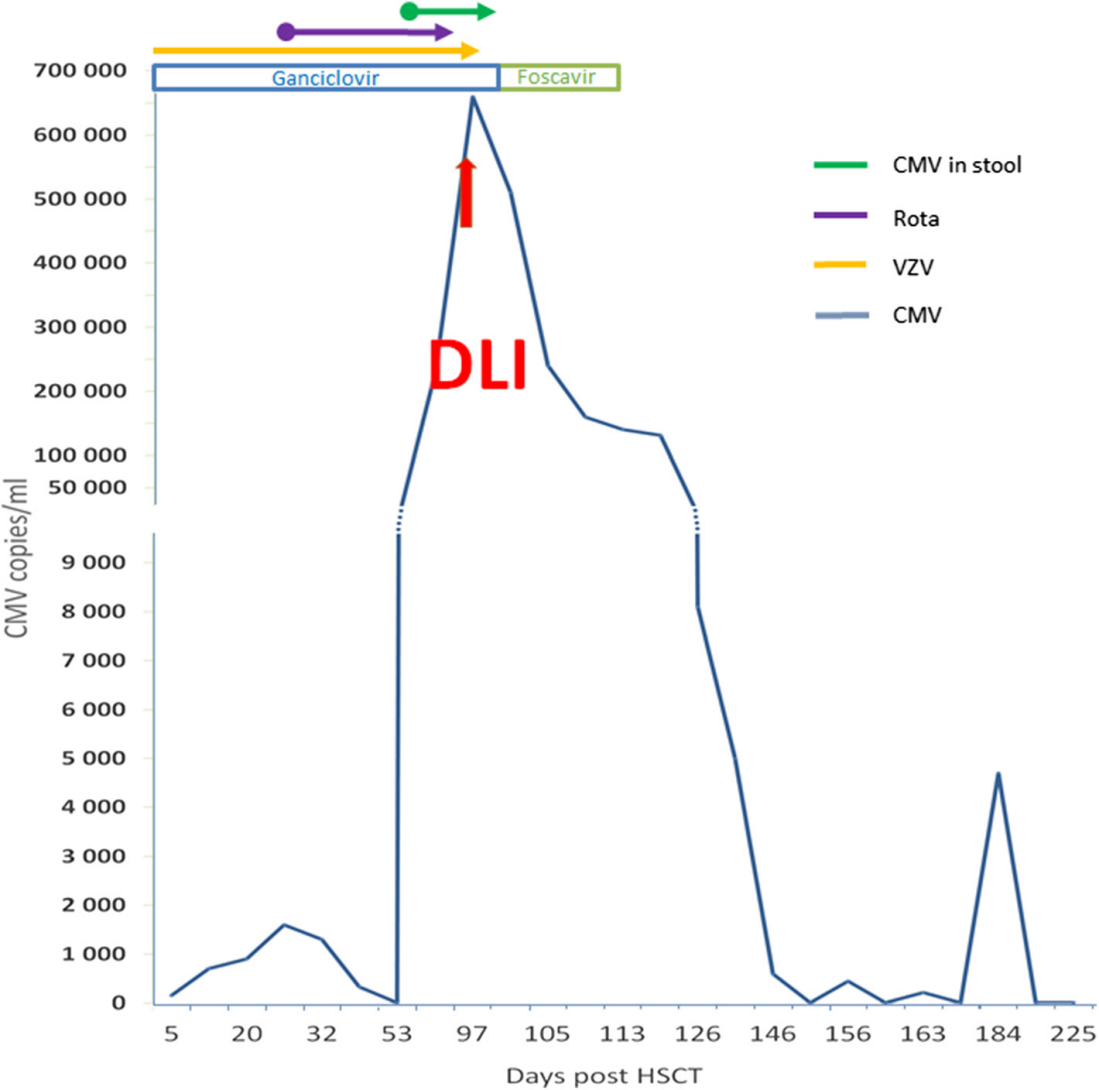
Viral load and antiviral treatment in relation to hematopoietic stem cell transplantation (HSCT) and donor lymphocyte infusion (DLI)

The patient did not experience any acute GvHD after HSCT or DLI. The early post-transplant period was complicated at day +5 by acute mechanical ileus with unknown etiology. This was resolved by mechanical manipulation during the exploratory laparotomy. The blood tests showed continued VZV positivity and the numbers of CMV copies were increasing. His condition deteriorated further and he suffered an *Enterococcus faecium* septicemia at day +74, followed by severe diarrhea and gastrointestinal tract bleeding, signs of probable colitis. The stool analysis revealed CMV, in addition to rotavirus. Two weeks after DLI, the test results showed that the CMV was resistant to ganciclovir; therefore, the antiviral treatment was changed to foscarnet. Following DLI, the time to clearance of VZV and rotavirus was 22 days. The CMV levels decreased rapidly (Fig. 2) and the patient’s condition improved considerably. The stool analysis for CMV was negative at 15 days following DLI and cleared from peripheral blood at 73 days. Supplementation with immunoglobulins was discontinued on day +99 post HSCT. He was discharged approximately 6 months post HSCT. No chronic GvHD was observed after the HSCT or DLI. He developed blindness and one-sided microphthalmia due to the varicella keratitis. At 21 months after HSCT, the patient had a normal lymphocyte count with normal lymphocyte subsets and neither new nor were exacerbations of preexisting infections observed.

## Discussion

Allogeneic HSCT is the only curative treatment available for SCID, although it is associated with a high incidence of severe infections and an increased risk of GvHD [2–4]. Early intervention is a crucial prognostic factor and the prompt availability of a parental donor may favor the HLA-haploidentical donor alternative. Haploidentical HSCT protocols utilizing extensively *ex vivo* T-cell depleted grafts have proven efficient in preventing GvHD [6, 7]. The remaining challenge of enhancing post-transplant immune-reconstitution has been addressed by several investigators by utilizing partially T-cell depleted grafts [10, 11, 21–23] or adoptive transfer of donor immune cells [24–26]. Here, we present a novel approach for treating SCID, combining TCR-α/β-cell depleted haploidentical HSCT with CD45RA+ depleted DLI for a therapeutic antiviral boost.

Our patient received a TCR-α/β-cell depleted graft from his haploidentical mother, following a reduced intensity conditioning regimen with a short course of ATG, a single dose of rituximab to prevent EBV-PTLD, and no additional GvHD prophylaxis. Engraftment was rapid with complete donor chimerism and no signs of GvHD. However, at 12 weeks post HSCT, the patient was still T-cell lymphopenic with clinical symptoms of multiple severe viral infections. Consequently, the decision was made to initiate therapeutic DLIs for enhanced anti-viral immunity.

In the haploidentical setting, unselected DLIs with a T-cell add-back of only 25 × 10^3^ CD3+ cells/kg may cause severe GvHD [6, 7]. In contrast, enriched CD3 + CD45RO+ memory T-cells may provide viral immunity with markedly reduced alloreactivity [13, 14]. Notwithstanding, cross-reactivity of memory viral-specific T-cells with mismatched HLA has been described and is, thus, a potential source of GvHD in haploidentical HSCT [27]. Based on these observations, the patient was treated with CD45RA+ depleted haploidentical donor lymphocytes, transferring a total T-cell dose of no more than 25 × 10^3^ CD3+ cells/kg with >99.9 % purity of CD3+ CD45RO+ memory T-cells. Following DLI, a prompt increase in CD3 + CD4+ and CD3 + CD8+ counts was observed, with a concomitant reduction in viral load and a subsequent clearance of viral infections. No acute or chronic GvHD was observed. As previously demonstrated [28], TCR-α/β cells could be efficiently depleted (log -5.0) with the CliniMACS instrument, preserving high numbers of CD34+ cells and potentially beneficial TCR-gd + cells and NK-cells in the graft. With the current approach of transferring a very low dose of CD45RA+ depleted donor T-cells (25 × 10^3^/kg CD3+ cells), a sample of donor peripheral blood will provide sufficient numbers of lymphocytes.

To ensure that the manufacturing of DLIs was in compliance with Good Manufacturing Process (GMP) conditions, a closed blood bag system was used for the collection and leukocyte enrichment of donor whole blood and the CliniMACS instrument was used for subsequent CD45RA+ depletion. Naïve T-cells (CD45RA+) were efficiently depleted (log -3.8) providing sufficient numbers of CD3 + CD45RO+ cells for the fresh DLI and for cryopreservation. A benefit of the current protocol is the time and cost effectiveness of using a whole blood donation rather than a leukapheresis product as starting material. Moreover, this approach is also less strenuous for the donor.

A long-standing approach to control refractory viral infections following HSCT is the generation of antigen-specific T-cells against single pathogens such as CMV, adenovirus, or EBV [29]. New strategies have been developed for *ex vivo* selection of pathogen-specific donor T-cells, using antigen induced stimulation and isolation by cytokine secretion assay [24] or HLA-peptide streptamers [25]. However, this approach is time consuming and technically much more demanding than the simple method of cell depletion based on surface CD45RA+ antigen expression. Also, the generation of antigen stimulated T-cells for clinical application is subjected to a more complex regulatory framework than that of CD45RA+ depleted, unmanipulated lymphocytes. Recently, the often limited therapeutic effect of using antigen-specific T-cells has been attributed to the limited in vivo persistence of transferred donor T-cells [29], suggesting that adoptive transfer should include a variety of donor T-cell populations to favor long-lasting clinical responses.

Very recently, three different groups [21, 22, 23] investigated the use of CD45RA+ depletion as part of the haploidentical or HLA-mismatched donor graft processing. Using different conditioning regimens with different stem cell sources and graft compositions, with or without serotherapy, two of the groups [21, 23] reported limited or no GvHD despite high numbers of transferred CD3 + CD45RO+ T-cells (1-100 x 10^6^/kg) while the third group reported a 17.6 % incidence of grade III/IV acute GvHD. Further studies are required to determine the optimal timing, T-cell dose, and source of CD45RA+ depleted donor lymphocytes - as part of the graft or as prophylactic, pre-emptive, or therapeutic DLIs.

## Conclusion

Automated depletion of CD45RA+ naïve T-cells from unmobilized whole blood donations is a simple and rapid strategy to provide unmanipulated DLIs, with a potentially broad repertoire of pathogen specific memory T-cells. In the haploidentical setting, therapeutic CD45RA+ depleted DLIs can be safely administered at low T-cell doses for efficient enhancement of viral immunity and limited risk of GvHD. We demonstrate the success of this approach following TCR-α/β-cell depleted HSCT for the treatment of SCID.

## Abbreviations

CMV: Cytomegalovirus
DLI: Donor lymphocyte infusion
DSA: HLA-donor specific antibodies
EBV-PTLD: Epstein–Barr virus-associated lymphoproliferative disease
GvHD: Graft-versus host-disease
HLA: Human leukocyte antigen
HSCT: Hematopoietic stem cell transplantation
MIP: Mutation Identification Pipeline
PBPCs: Peripheral blood progenitor cells
PID: Primary immunodeficiency disorder
SCID: Severe combined immunodeficiency
TCR: T cell receptor
URTI: Upper respiratory tract infection
VZV: varicella zoster virus

## References

[1] van der Burg M, Gennery AR (2011). Educational paper. The expanding clinical and immunological spectrum of severe combined immunodeficiency. Eur J Pediatr.

[2] Brown L, Xu-Bayford J, Allwood Z, Slatter M, Cant A, Davies EG (2011). Neonatal diagnosis of severe combined immunodeficiency leads to significantly improved survival outcome: the case for newborn screening. Blood.

[3] Dvorak CC, Cowan MJ, Logan BR, Notarangelo LD, Griffith LM, Puck JM (2013). The natural history of children with severe combined immunodeficiency: baseline features of the first fifty patients of the primary immune deficiency treatment consortium prospective study 6901. J Clin Immunol.

[4] Pai SY, Logan BR, Griffith LM, Buckley RH, Parrott RE, Dvorak CC (2014). Transplantation outcomes for severe combined immunodeficiency, 2000-2009. N Engl J Med.

[5] Gaspar HB, Qasim W, Davies EG, Rao K, Amrolia PJ, Veys P (2013). How I treat severe combined immunodeficiency. Blood.

[6] Handgretinger R, Klingebiel T, Lang P, Schumm M, Neu S, Geiselhart A (2001). Megadose transplantation of purified peripheral blood CD34(+) progenitor cells from HLA-mismatched parental donors in children. Bone Marrow Transplant.

[7] Klingebiel T, Handgretinger R, Lang P, Bader P, Niethammer D (2004). Haploidentical transplantation for acute lymphoblastic leukemia in childhood. Blood Rev.

[8] Lang P, Greil J, Bader P, Handgretinger R, Klingebiel T, Schumm M (2004). Long-term outcome after haploidentical stem cell transplantation in children. Blood Cells Mol Dis.

[9] Lang P, Teltschik HM, Feuchtinger T, Muller I, Pfeiffer M, Schumm M (2014). Transplantation of CD3/CD19 depleted allografts from haploidentical family donors in paediatric leukaemia. Br J Haematol.

[10] Bertaina A, Merli P, Rutella S, Pagliara D, Bernardo ME, Masetti R (2014). HLA-haploidentical stem cell transplantation after removal of alphabeta + T and B cells in children with nonmalignant disorders. Blood.

[11] Lang P, Feuchtinger T, Teltschik HM, Schwinger W, Schlegel P, Pfeiffer M (2015). Improved immune recovery after transplantation of TCRalphabeta/CD19-depleted allografts from haploidentical donors in pediatric patients. Bone Marrow Transplant.

[12] Mahnke YD, Brodie TM, Sallusto F, Roederer M, Lugli E (2013). The who's who of T-cell differentiation: human memory T-cell subsets. Eur J Immunol.

[13] Bleakley M, Heimfeld S, Jones LA, Turtle C, Krause D, Riddell SR (2014). Engineering human peripheral blood stem cell grafts that are depleted of naive T cells and retain functional pathogen-specific memory T cells. Biol Blood Marrow Transplant.

[14] Teschner D, Distler E, Wehler D, Frey M, Marandiuc D, Langeveld K (2014). Depletion of naive T cells using clinical grade magnetic CD45RA beads: a new approach for GVHD prophylaxis. Bone Marrow Transplant.

[15] Brodszki N, Jonsson G, Skattum L, Truedsson L (2014). Primary immunodeficiency in infection-prone children in southern Sweden: occurrence, clinical characteristics and immunological findings. BMC Immunol.

[16] Stranneheim H, Engvall M, Naess K, Lesko N, Larsson P, Dahlberg M (2014). Rapid pulsed whole genome sequencing for comprehensive acute diagnostics of inborn errors of metabolism. BMC Genomics.

[17] Stern M, Ruggeri L, Mancusi A, Bernardo ME, de Angelis C, Bucher C (2008). Survival after T cell-depleted haploidentical stem cell transplantation is improved using the mother as donor. Blood.

[18] ESID/EBMT. EBMT/ESID guidelines for haematopoietic stem cell transplantation for primary immunodeficiencies. 2011.

[19] Oevermann L, Lang P, Feuchtinger T, Schumm M, Teltschik HM, Schlegel P (2012). Immune reconstitution and strategies for rebuilding the immune system after haploidentical stem cell transplantation. Ann N Y Acad Sci.

[20] Podgorny PJ, Ugarte-Torres A, Liu Y, Williamson TS, Russell JA, Storek J (2010). High rabbit-antihuman thymocyte globulin levels are associated with low likelihood of graft-vs-host disease and high likelihood of posttransplant lymphoproliferative disorder. Biol Blood Marrow Transplant.

[21] Shook DR, Triplett BM, Eldridge PW, Kang G, Srinivasan A, Leung W (2015). Haploidentical stem cell transplantation augmented by CD45RA negative lymphocytes provides rapid engraftment and excellent tolerability. Pediatr Blood Cancer.

[22] Triplett BM, Shook DR, Eldridge P, Li Y, Kang G, Dallas M (2015). Rapid memory T-cell reconstitution recapitulating CD45RA-depleted haploidentical transplant graft content in patients with hematologic malignancies. Bone Marrow Transplant.

[23] Touzot F, Neven B, Dal-Cortivo L, Gabrion A, Moshous D, Cros G et al. CD45RA depletion in HLA-mismatched allogeneic hematopoietic stem cell transplantation for primary combined immunodeficiency: A preliminary study. J Allergy Clin Immunol. 2015;135(5):1303-9 e1-3. 10.1016/j.jaci.2014.08.019 25282016

[24] Feuchtinger T, Opherk K, Bethge WA, Topp MS, Schuster FR, Weissinger EM (2010). Adoptive transfer of pp 65-specific T cells for the treatment of chemorefractory cytomegalovirus disease or reactivation after haploidentical and matched unrelated stem cell transplantation. Blood.

[25] Schmitt A, Tonn T, Busch DH, Grigoleit GU, Einsele H, Odendahl M (2011). Adoptive transfer and selective reconstitution of streptamer-selected cytomegalovirus-specific CD8+ T cells leads to virus clearance in patients after allogeneic peripheral blood stem cell transplantation. Transfusion.

[26] Martelli MF, Di Ianni M, Ruggeri L, Falzetti F, Carotti A, Terenzi A (2014). HLA-haploidentical transplantation with regulatory and conventional T-cell adoptive immunotherapy prevents acute leukemia relapse. Blood.

[27] Melenhorst JJ, Scheinberg P, Williams A, Ambrozak DR, Keyvanfar K, Smith M (2011). Alloreactivity across HLA barriers is mediated by both naive and antigen-experienced T cells. Biol Blood Marrow Transplant.

[28] Dykes JH HA, Toporski J, Lenhoff S, Scheding S, Turkiewicz D (2013). Effective TcR α/β+ cell depletion using the CliniMACS® system to produce peripheral blood progenitor cell products for haploidentical transplantation. Bone Marrow Transplant.

[29] Einsele H, Loffler J, Kapp M, Rasche L, Mielke S, Grigoleit UG (2015). Immunotherapy for viral and fungal infections. Bone Marrow Transplant.

